# Gaussian Correlation via Inverse Brascamp–Lieb

**DOI:** 10.1007/s00440-025-01445-x

**Published:** 2025-11-13

**Authors:** Emanuel Milman

**Affiliations:** Department of Mathematics, Technion Israel Institute of Technology, Haifa, 32000 Israel

**Keywords:** Gaussian correlation inequality, Inverse Brascamp–Lieb inequality, Even log-concave functions, 52A40, 60E15, 60G15

## Abstract

We give a simple alternative proof of Royen’s Gaussian Correlation inequality by using (a slightly generalized version of) Nakamura–Tsuji’s symmetric inverse Brascamp–Lieb inequality for even log-concave functions. We explain that this inverse inequality is in a certain sense a dual counterpart to the forward inequality of Bennett–Carbery–Christ–Tao and Valdimarsson, and that the log-concavity assumption therein cannot be omitted in general.

## Introduction

Our starting point in this note is the observation that centered Gaussians saturate the following correlation lower-bound. This phenomenon (for the corresponding upper bound) has its origins in the work of Brascamp and Lieb [11]. Given a symmetric n×n matrix *A*, we denote $g_{A}(x){:}{=} \exp (-\frac{1}{2} \left\langle A x, x \right\rangle )$ for $x \in \mathbb {R}^n$; when A>0 is positive-definite, this corresponds to a centered Gaussian (whose covariance, after normalizing $g_A$ to be a probability density, is $A^{-1}$). All functions below are assumed to be non-negative. Recall that a function $f: \mathbb {R}^n \rightarrow \mathbb {R}_+$ is called log-concave (respectively, log-convex) if $\log f: \mathbb {R}^n \rightarrow \mathbb {R}\cup \{-\infty \}$ is concave (respectively, convex). Let $n_1,\ldots ,n_m$ be positive integers, set $N = \sum _{i=1}^m n_i$, and consider the orthogonal decomposition $\mathbb {R}^N = \oplus _{i=1}^m \mathbb {R}^{n_i}$. Given $x \in \mathbb {R}^N$, write $x = (x_1,\ldots ,x_m)$ with $x_i \in \mathbb {R}^{n_i}$.

### Theorem 1.1

(Symmetric Inverse Brascamp–Lieb Inequality, after Nakamura–Tsuji [33]) Let $\bar{Q}$ be an N×N symmetric matrix (**of arbitrary signature**), and let $Q_i \ge 0$ denote positive semi-definite $n_i \times n_i$ matrices, for i=1,…,m. Let $c_1,\ldots ,c_m>0$. Then for all (non-zero) **even log-concave** $h_i \in L^1(\mathbb {R}^{n_i},g_{Q_i}(x_i) dx_i)$, i=1,…,m, we have:

$$\begin{aligned} \frac{\int _{\mathbb {R}^N} e^{-\frac{1}{2}\left\langle \bar{Q} x,x \right\rangle } \Pi _{i=1}^m h_i(x_i)^{c_i} dx}{\Pi _{i=1}^m \left( \int _{\mathbb {R}^{n_i}} e^{-\frac{1}{2} \left\langle Q_i x_i, x_i \right\rangle } h_i(x_i) dx_i\right) ^{c_i} } \ge \inf _{\begin{array}{c} A_i \ge 0\\ A_i+Q_i > 0 \end{array} } \frac{\int _{\mathbb {R}^N} e^{-\frac{1}{2} \left\langle \bar{Q} x,x \right\rangle } \Pi _{i=1}^m g_{A_i}(x_i)^{c_i} dx}{\Pi _{i=1}^m \left( \int _{\mathbb {R}^{n_i}} e^{-\frac{1}{2} \left\langle Q_i x_i, x_i \right\rangle } g_{A_i}(x_i) dx_i\right) ^{c_i} } , \end{aligned}$$

where the infimum is over $n_i \times n_i$ symmetric matrices $A_i$, i=1,…,m.

Theorem 1.1 was recently established for $Q_i = 0$ by Nakamura and Tsuji in [33, Theorem 1.3]. The extended version for general $Q_i \ge 0$ above is obtained by a straightforward repetition of their proof, which we verify in Section 2. We refer to [33] for a thorough discussion and comparison with the original inequalities of Brascamp–Lieb and Lieb [11, 29] (see also Carlen–Lieb–Loss [16], Bennett–Carbery–Christ–Tao [8]), the reverse Brascamp–Lieb inequality of Barthe [2, 3] (see also Barthe–Cordero-Erausquin [4], Valdimarsson [41] and Barthe–Huet [6]), the inverse Brascamp–Lieb inequalities of Chen–Dafnis–Paouris [17] and Barthe–Wolff [7], and the unified forward-reverse Brascamp–Lieb inequalities of Liu–Courtade–Cuff–Verdú [20, 30]. See also [5, 10, 15] and the references therein for further related results. When $Q_i=0$ and $\bar{Q}=0$, scale invariance clearly implies that one should assume $\sum _{i=1}^m c_i n_i = N$ in order for the left-hand-side to have any chance of being bounded away from 0 and ∞, but this is not the case for general $Q_i$ or $\bar{Q}$. The above mentioned works treat more general surjective linear transformations $P_i: \mathbb {R}^N \rightarrow \mathbb {R}^{n_i}$ with $\cap _{i=1}^m \ker P_i = \{ 0\}$, but we will only require and treat the case when $P_i x{:}{=} x_i$ for $x = (x_1,\ldots ,x_m)$, as in [33]. Here and below $Q_1 \oplus \ldots \oplus Q_m$ denotes the block matrix $P^*_1 Q_1 P_1 + \ldots + P^*_m Q_m P_m$.

We remark that some additional partial cases of Theorem 1.1 were previously known in the literature. Denoting

1.1 $$\begin{aligned} Q {:}{=} \bar{Q} - c_1 Q_1 \oplus \ldots \oplus c_m Q_m, \end{aligned}$$

the case that Q=0 and general $Q_i > 0$ without requiring any evenness is contained in [41, Theorem 1.4 (2)] of Valdimarsson, and the case when Q≤0 without requiring evenness nor log-concavity of $h_i$ on the left but also without requiring that $A_i \ge 0$ on the right is contained in [7, Theorem 1.4] of Barthe–Wolff (see also Chen–Dafnis–Paouris [17] for the particular “geometric case" when in addition $P_i \bar{Q}^{-1} P_i^* = Q_i^{-1}$ for all *i* and $\bar{Q} > 0$); however, neither of these assumptions fits our purposes.

### Log-concavity assumption

A noteworthy feature of the Nakamura–Tsuji formulation is that, contrary to the inverse Brascamp–Lieb inequalities in [7, 17], there is no restriction on the signature of *Q*. As explained in [7, 33], the price for this flexibility is that the test functions $f_i$ cannot be arbitrarily translated, and are therefore assumed to be even. In [33], Nakamura and Tsuji further discuss whether the additional assumption of log-concavity is also natural (as it is absent from the formulations in [7, 17, 31, 32]), and write that it is actually reasonable to expect that it could be omitted. However, we shall see below that Theorem 1.1 would be false without the log-concavity assumption (at least, if one allows considering $Q_i > 0$). Furthermore, the log-concavity assumption is actually natural, as we shall now attempt to explain.

Given a positive semi-definite $Q_i \ge 0$, we shall say $f_i$ is more log-concave (respectively, more log-convex) than $g_{Q_i}$ if $f_i = g_{Q_i} h_i$ for some log-concave (respectively, log-convex) function $h_i$. We shall say that $f_i$ is generated by $g_{Q_i}$ (“of $Q_i$-type" in the terminology of [8]) if $f_i$ is obtained as the convolution $g_{Q_i} *\mu _i$ for some finite (non-zero) Borel measure $\mu _i$ (note that such $f_i$ is always smooth). For smooth and positive $f_i$, clearly $f_i$ is more log-concave (respectively, more log-convex) than $g_{Q_i}$ iff $\text {Hess}(-\log f_i) \ge (\le ) \, Q_i$, and it is easy to check that if $f_i$ is generated by $g_{Q_i}$ then it is more log-convex than $g_{Q_i}$ (e.g. [18, Lemma 16]). Clearly, recalling the definition (1.1) of *Q* and setting $B_i{:}{=} A_i + Q_i$ and $f_i = g_{Q_i} h_i$, Theorem 1.1 may be equivalently reformulated as follows (note that the equivalence crucially relies on the signatures of $\bar{Q}$ and *Q* being arbitrary):

#### Theorem 1.2

(Theorem 1.1 again) Let *Q* be an N×N symmetric matrix (**of arbitrary signature**), and let $Q_i \ge 0$ denote positive semi-definite $n_i \times n_i$ matrices, for i=1,…,m. Let $c_1,\ldots ,c_m>0$. Then for all (non-zero) **even** $f_i \in L^1(\mathbb {R}^{n_i})$ which are **more log-concave than** $g_{Q_i}$, we have:

$$ \frac{\int _{\mathbb {R}^N} e^{-\frac{1}{2}\left\langle Q x,x \right\rangle } \Pi _{i=1}^m f_i(x_i)^{c_i} dx}{\Pi _{i=1}^m \left( \int _{\mathbb {R}^{n_i}} f_i(x_i) dx_i\right) ^{c_i} } \ge \inf _{\begin{array}{c} B_i \ge Q_i\\ B_i > 0 \end{array}} \frac{\int _{\mathbb {R}^N} e^{-\frac{1}{2} \left\langle Q x,x \right\rangle } \Pi _{i=1}^m g_{B_i}(x_i)^{c_i} dx}{\Pi _{i=1}^m \left( \int _{\mathbb {R}^{n_i}} g_{B_i}(x_i) dx_i\right) ^{c_i} } . $$

On the other hand, the following forward generalized Brascamp–Lieb inequality was established by Bennett–Carbery–Christ–Tao [8, Corollaries 8.15 and 8.16] and Valdimarsson [41, Theorem 1.4] whenever $f_i$ are generated by $g_{Q_i}$. Valdimarsson’s proof is based on a generalized version of Caffarelli’s contraction theorem [14], stating that if $f_i$ is generated by $g_{Q_i}$ and $h_i$ is more log-concave than $g_{Q_i^{-1}}$, then Brenier’s optimal transport map $T = \nabla \varphi$ [13] pushing forward $\mu = a f_i(x) dx$ onto $\nu = b h_i(x) dx$ (both normalized to be probability measures) satisfies $0 \le \text {Hess}\; \varphi \le Q_i$ [41, Theorem 1.2]. In fact, the assumption that $f_i$ is generated by $g_{Q_i}$ can be relaxed to the assumption that $f_i$ is more log-convex than $g_{Q_i}$. This follows from an extension of Caffarelli’s contraction theorem due to Kolesnikov [27, Theorem 2.2], who showed that if $f^0_i$ is more log-convex than $g_{\text {Id}}$ and $h^0_i$ is more log-concave than $g_{\text {Id}}$ then the Brenier map $T_0 = \nabla \varphi _0$ pushing forward $\mu _0 = a f_i^0(x) dx$ onto $\nu _0 = b h_i^0(x) dx$ satisfies $0 \le \text {Hess}\; \varphi _0\le \text {Id}$; applying this to $\mu _0$ and $\nu _0$ given by the push-forwards of μ by $\sqrt{Q_i}$ and ν by $(\sqrt{Q_i})^{-1}$, respectively, and setting $\varphi = \varphi _0 \circ \sqrt{Q_i}$, it follows that $T = \nabla \varphi = \sqrt{Q_i} \circ T_0 \circ \sqrt{Q_i}$ is the Brenier map pushing forward μ onto ν, and it satisfies $0 \le \text {Hess}\; \varphi \le Q_i$. See also the recent extensions to the case when $f_i$ is more log-convex than $g_{A_i}$ and $h_i$ is more log-concave than $g_{B_i^{-1}}$ for commuting and even general $A_i,B_i > 0$ due to Chewi–Pooladian [18, Theorem 13] and Gozlan–Sylvestre [23, Theorem 5.4], respectively. Repeating Valdimarsson’s argument from [41], the following is thereby immediately deduced (see the proof of [8, Lemma 8.13] to reduce to the case Q=0):

#### Theorem 1.3

(After [8, 27, 41]) Let Q≥0 be an N×N **positive semi-definite** matrix, and let $Q_i \ge 0$ denote positive semi-definite $n_i \times n_i$ matrices, for i=1,…,m. Let $c_1,\ldots ,c_m>0$. Then for all (non-zero) $f_i \in L^1(\mathbb {R}^{n_i})$ which are **more log-convex than** $g_{Q_i}$ (but not necessarily even),

$$ \frac{\int _{\mathbb {R}^N} e^{-\frac{1}{2}\left\langle Q x,x \right\rangle } \Pi _{i=1}^m f_i(x_i)^{c_i} dx}{\Pi _{i=1}^m \left( \int _{\mathbb {R}^{n_i}} f_i(x_i) dx_i\right) ^{c_i} } \le \inf _{0 < B_i \le Q_i} \frac{\int _{\mathbb {R}^N} e^{-\frac{1}{2}\left\langle Q x,x \right\rangle } \Pi _{i=1}^m g_{B_i}(x_i)^{c_i} dx}{\Pi _{i=1}^m \left( \int _{\mathbb {R}^{n_i}} g_{B_i}(x_i) dx_i\right) ^{c_i} } . $$

Taking $Q_i = \Lambda \cdot \text {Id}_{n_i}$ with $\Lambda \nearrow +\infty$, one (formally) recovers a result of Lieb [29, Theorem 6.2] (see also [8, Corollary 8.16]), in which $f_i \in L^1(\mathbb {R}^{n_i})$ are arbitrary. See also [9, Theorem 2.1] for an inverse Brascamp–Lieb inequality for functions generated by $g_{Q_i}$. In addition, Valdimarsson established in [41, Theorem 1.4 (2)] an analogous *reverse* Brascamp–Lieb inequality for the sup-convolution of functions $f_i$ which are more log-concave than $g_{Q_i}$, extending Barthe’s proof from [3]; as already mentioned, when $P_i(x) = x_i$, this reverse form coincides with the case Q=0 and $Q_i > 0$ of Theorem 1.2, but without requiring the evenness assumption. In view of these analogies (which seem to hint at a natural duality relation between the forward and inverse directions), the requirement that $h_i$ be log-concave in Theorem 1.1 is very natural.

### Gaussian Correlation Inequality

Our main motivation for insisting on the extended version of Theorem 1.1 is that it leads to a new and simple proof of Royen’s Gaussian Correlation Inequality [36] (see also [28]):

#### Theorem 1.4

(Gaussian Correlation Inequality [36]) Let $X = (X_1,\ldots ,X_N)$ denote a centered Gaussian random vector in $\mathbb {R}^N$ (with arbitrary positive-definite covariance). Then for all $n_1 + n_2 = N$:

$$ \mathbb {P}(\max _{1 \le i \le N} |X_i| \le 1) \ge \mathbb {P}(\max _{1 \le i \le n_1} |X_i| \le 1) \; \mathbb {P}(\max _{n_1+1 \le i \le N} |X_i| \le 1) . $$

The case $\min (n_1,n_2)=1$ above was previously established independently by Khatri [26] and Šidák [42]; see also [19, 24, 39] for additional prior results and [1, 22, 37, 40] for extensions. In fact, Royen established this for a more general family of Gamma distributions, but we restrict our attention to the Gaussian case, for which the non-negative correlation was originally investigated by Pitt [35] and remained open (in general) for several decades. Since the inequality holds for arbitrary dimension *N* and arbitrary covariance, it is well-known and easy to verify (see e.g. [28, 39] or Subsection 3.3) that it is equivalent to any of the following statements. Recall that $P_i: \mathbb {R}^N \rightarrow \mathbb {R}^{n_i}$ is given by $P_i x = x_i$ for $x = (x_1,x_2) \in \mathbb {R}^{n_1} \oplus \mathbb {R}^{n_2}$.

- For all origin-symmetric convex sets K=-K and L=-L in $\mathbb {R}^n$ and all n≥1:
  $$ \gamma ^n(K \cap L) \ge \gamma ^n(K) \gamma ^n(L) , $$
  where $\gamma ^n$ denotes the standard Gaussian measure on $\mathbb {R}^n$. This formulation was established for n=2 by Pitt [35].
- For all even quasi-concave functions $f_1,f_2: \mathbb {R}^{N} \rightarrow \mathbb {R}_+$, namely functions whose super-level sets $\{ f_i \ge a\}$ are origin-symmetric convex sets for all *a*, one has:
  $$ \mathbb {E}f_1(X) f_2(X) \ge \mathbb {E}f_1(X) \mathbb {E}f_2(X) . $$
- For all even quasi-concave functions $f_i: \mathbb {R}^{n_i} \rightarrow \mathbb {R}_+$, i=1,2, one has:
  $$ \mathbb {E}f_1(P_1 X) f_2(P_2 X) \ge \mathbb {E}f_1(P_1 X) \mathbb {E}f_2(P_2 X) . $$

To see that Theorem 1.1 yields Theorem 1.4, denote by Σ, $\Sigma _1$ and $\Sigma _2$ the covariance matrices of *X*, $P_1 X$ and $P_2 X$, respectively. Applying Theorem 1.1 with $\bar{Q} = \Sigma ^{-1}$, $Q_1 = \Sigma _1^{-1}$, $Q_2 = \Sigma _2^{-1}$, $c_1 = c_2 = 1$, m=2 and the even log-concave $h_i(x_i) = \textbf{1}_{K_i}(x_i)$ with $K_i = [-1,1]^{n_i}$, we immediately obtain:

1.2 $$\begin{aligned}&\frac{\mathbb {P}(\max _{1 \le i \le N} |X_i| \le 1) }{\mathbb {P}(\max _{1 \le i \le n_1} |X_i| \le 1) \; \mathbb {P}(\max _{n_1+1 \le i \le N} |X_i| \le 1)} \nonumber \\&= \frac{ \mathbb {E}\textbf{1}_{K_1}(P_1 X) \textbf{1}_{K_2}(P_2 X) }{\mathbb {E}\textbf{1}_{K_1}(P_1 X) \mathbb {E}\textbf{1}_{K_2}(P_2 X)} \ge \inf _{A_1,A_2 \ge 0} \frac{\mathbb {E}g_{A_1}(P_1 X) g_{A_2}(P_2 X)}{\mathbb {E}g_{A_1}(P_1 X) \mathbb {E}g_{A_2}(P_2 X)} \end{aligned}$$

1.3 $$\begin{aligned}&= \inf _{A_1,A_2 \ge 0} \left( \frac{\det (\text {Id}_{n_1} + A_1 \Sigma _1) \det (\text {Id}_{n_2} + A_2 \Sigma _2) }{ \det (\text {Id}_{N} + (A_1 \oplus A_2) \Sigma )}\right) ^{\frac{1}{2}} \end{aligned}$$

1.4 $$\begin{aligned}&= \inf _{A_1,A_2 \ge 0} \left( \frac{\det (\text {Id}_{n_1} + \sqrt{A_1} \Sigma _1 \sqrt{A_1}) \det (\text {Id}_{n_2} + \sqrt{A_2} \Sigma _2 \sqrt{A_2}) }{ \det (\text {Id}_{N} + \sqrt{A_1 \oplus A_2} \Sigma \sqrt{A_1 \oplus A_2})}\right) ^{\frac{1}{2}} , \end{aligned}$$

where in the last transition we used the obvious fact that $\det (\text {Id}_n + A B) = \det (\text {Id}_n + BA)$ for all n×n matrices *A*, *B*.

It remains to show that the infimum in (1.4) is precisely 1, attained at $A_1 = A_2 = 0$. This follows from the following inequality of Fischer [25, Theorem 7.8.5], which is an equivalent version of a classical inequality of Hadamard [25, Theorem 7.8.1]:

#### Lemma 1.5

(Fischer’s inequality) Let $$M = \begin{pmatrix} M_1 &  M_o\\ M_o^* &  M_2 \end{pmatrix}$$ denote a positive-definite Hermitian matrix in block form. Then:

1.5 $$\begin{aligned} \det (M) \le \det (M_1) \det (M_2) , \end{aligned}$$

with equality if and only if $M_o = 0$.

Writing $$\Sigma = \begin{pmatrix} \Sigma _1 &  \Sigma _o \\ \Sigma _o^* &  \Sigma _2 \end{pmatrix}$$ and applying (1.5) to the positive-definite matrix

1.6 $$\begin{aligned} \text {Id}_N + \sqrt{A_1 \oplus A_2} \Sigma \sqrt{A_1 \oplus A_2} = \begin{pmatrix} \text {Id}_{n_1} + \sqrt{A_1} \Sigma _1\sqrt{A_1} &  \sqrt{A_1} \Sigma _o \sqrt{A_2} \\ \sqrt{A_2} \Sigma _o^* \sqrt{A_1} &  \text {Id}_{n_2} + \sqrt{A_2} \Sigma _2 \sqrt{A_2} \end{pmatrix} , \end{aligned}$$

this concludes the proof of Theorem 1.4. The above simple argument for showing that the infimum in (1.4) is equal to 1 is due to an anonymous referee, whom we would like to thank. Our original argument relied on a monotonicity lemma established by Royen in [36] – see Section 3 for a comparison between our approach and Royen’s one, including analysis of the equality case in Theorem 1.4.

The above reasoning in fact shows that the log-concavity assumption in Theorem 1.1 cannot be omitted. Otherwise, the same proof would imply that:

$$ \mathbb {P}( P_1 X \in K \wedge P_2 X \in L) \ge \mathbb {P}(P_1 X \in K) \; \mathbb {P}(P_2 X \in L) , $$

for any origin-symmetric $K=-K \subset \mathbb {R}^{n_1}$ and $L=-L \subset \mathbb {R}^{n_2}$ regardless of convexity. Applying this to both *L* and its complement and summing, it would follow that we must actually have equality above. But clearly it is not true that

$$ \mathbb {P}( P_1 X \in K \wedge P_2 X \in L) = \mathbb {P}(P_1 X \in K) \; \mathbb {P}(P_2 X \in L) $$

for general covariance matrices Σ>0 even when *K* and *L* slabs. Consequently, we deduce (for example) that Theorem 1.1 is false for N=2, m=2, $n_1=n_2=1$, $h_1 = {\textbf {1}}_{[-1,1]}$, $h_2 = {\textbf {1}}_{\mathbb {R}\setminus [-1,1]}$ (which is not log-concave), $\bar{Q} = \Sigma ^{-1}$, $Q_i = \Sigma _{i}^{-1}$ and $c_1=c_2=1$, for generic positive-definite 2×2 matrices Σ.

## Proof of Theorem 1.2

The proof of Theorem 1.2 is a verbatim repetition of the proof of [33, Theorem 1.3] by Nakamura–Tsuji. Note that we will use the formulation of Theorem 1.2 rather than the one of the equivalent Theorem 1.1. Consequently, even if one is only interested in the case $\bar{Q} \ge 0$ of Theorem 1.1, we must handle *Q* of arbitrary signature in Theorem 1.2 to make the equivalence valid.

Let us now sketch the proof of Theorem 1.2, emphasizing the necessary adjustments for handling general $Q_i \ge 0$. Given a sequence of positive semi-definite $n_i \times n_i$ matrices $A_i \ge 0$, i=1,…,m, denote $\textbf{A}= (A_1,\ldots ,A_m)$, writing $\textbf{A}\ge 0$. When $A_i = \lambda \text {Id}_{n_i}$ for all i=1,…,m, we will write $\textbf{A}= \lambda \text {Id}$. We will also denote $\textbf{A}+\textbf{B}= (A_i + B_i)_{i=1,\ldots ,m}$, and write $\textbf{A}> 0$ or $\textbf{A}\le \textbf{B}$ if $A_i > 0$ or $A_i \le B_i$ for all i=1,…,m, respectively. Given $0 \le \textbf{A}\le \textbf{B}$, denote by $\mathcal {F}_{\textbf{A}}$ the collection of $\textbf{f}= (f_1,\ldots ,f_m)$ so that $f_i \in L^1(\mathbb {R}^{n_i}, \mathbb {R}_+)$ is even, non-zero, and more log-concave than $g_{A_i}$ for all i=1,…,m, and by $\mathcal {F}_{\textbf{A},\textbf{B}} \subset \mathcal {F}_\textbf{A}$ the subfamily so that $f_i$ is in addition more log-convex than $g_{B_i}$. Also denote by $\mathcal {G}_\textbf{A}\subset \mathcal {F}_\textbf{A}$ (respectively, $\mathcal {G}_{\textbf{A},\textbf{B}} \subseteq \mathcal {F}_{\textbf{A},\textbf{B}}$) the subfamilies consisting of centered Gaussians, namely $\textbf{g}= (g_1,\ldots ,g_m)$ where $g_i = g_{C_i}$ with $C_i > 0$ and $C_i \ge A_i$ (respectively, $A_i \le C_i \le B_i$).

Now fix *Q* and $c_i > 0$ as in Theorem 1.2, set:

$$ \mathcal{B}\mathcal{L}(\textbf{f}) {:}{=} \frac{\int e^{-\frac{1}{2}\left\langle Q x,x \right\rangle } \Pi _{i=1}^m f_i(x_i)^{c_i} dx}{\Pi _{i=1}^m \left( \int _{\mathbb {R}^{n_i}} f_i(x_i) dx_i\right) ^{c_i} } , $$

and denote:

$$ I_{\textbf{A},\textbf{B}} {:}{=} \inf _{\textbf{f}\in \mathcal {F}_{\textbf{A},\textbf{B}}} \mathcal{B}\mathcal{L}(\textbf{f}) ~,~ I_{\textbf{A}} {:}{=} \inf _{\textbf{f}\in \mathcal {F}_{\textbf{A}}} \mathcal{B}\mathcal{L}(\textbf{f}) ~,~ I^{\mathcal {G}}_{\textbf{A},\textbf{B}} {:}{=} \inf _{\textbf{g}\in \mathcal {G}_{\textbf{A},\textbf{B}}} \mathcal{B}\mathcal{L}(\textbf{g}) ~,~ I^{\mathcal {G}}_{\textbf{A}} {:}{=} \inf _{\textbf{g}\in \mathcal {G}_{\textbf{A}}} \mathcal{B}\mathcal{L}(\textbf{g}) . $$

The proof of Theorem 1.2 consists of the following four steps. Fix $0 < \textbf{A}\le \textbf{B}$ (we may assume that $\textbf{B}= \Lambda \text {Id}$).

1. The infimum in the definition of $I_{\textbf{A},\textbf{B}}$ is attained on some $\textbf{f}\in \mathcal {F}_{\textbf{A},\textbf{B}}$.
2. If $$f \in \mathcal {F}_{\textbf{A},\textbf{B}}$$ then
  $$ \mathcal{B}\mathcal{L}(\textbf{f})^2 \ge I_{\textbf{A},\textbf{B}} \, \mathcal{B}\mathcal{L}({{\,\mathrm{\text {Conv}}\,}}\textbf{f}), $$
  where $${{\,\mathrm{\text {Conv}}\,}}\textbf{f}= ({{\,\mathrm{\text {Conv}}\,}}f_1,\ldots ,{{\,\mathrm{\text {Conv}}\,}}f_m)$$ and $${{\,\mathrm{\text {Conv}}\,}}f_i := 2^{n_i/2} (f_i *f_i)(\sqrt{2} \; \cdot )$$.
3. $$I_{\textbf{A},\textbf{B}} \ge I^\mathcal {G}_{\textbf{A},\textbf{B}}$$ and hence $$I_{\textbf{A},\textbf{B}} = I^\mathcal {G}_{\textbf{A},\textbf{B}}$$.
4. For all $$\textbf{Q}\ge 0$$, $$I_\textbf{Q}= \lim _{\lambda \rightarrow 0^+} \lim _{\Lambda \rightarrow \infty } I_{\textbf{Q}+ \lambda \text {Id}, \Lambda \text {Id}}$$.

These steps were carried out in [33] for $\textbf{A}= \lambda \text {Id}$, $\textbf{B}= \Lambda \text {Id}$ and $\textbf{Q}= \lambda _0 \text {Id}$, $0 \le \lambda _0< \lambda \le \Lambda < \infty$. The only required modifications to the proof for handling general $\textbf{A},\textbf{B},\textbf{Q}$ as above are encapsulated in the following simple:

### Lemma 2.1

Let $$f_i \in L^1(\mathbb {R}^n,\mathbb {R}_+)$$, i=1,2.

(i) If $f_i$ is more log-concave (respectively, more log-convex) than $g_{A_i}$, $A_i > 0$ and i=1,2, then for all $x \in \mathbb {R}^n$, $\mathbb {R}^n \ni y \mapsto f_1(\frac{x+y}{\sqrt{2}}) f_2(\frac{x-y}{\sqrt{2}})$ is more log-concave (respectively, more log-convex) than $g_{\frac{A_1+A_2}{2}}$.
(ii) If $f_i$ is more log-concave (respectively, more log-convex) than $g_{A_i}$, $A_i > 0$ and i=1,2, then $f_1 *f_2$ is more log-concave (respectively, more log-convex) than $g_{C}$ for $C^{-1} = A_1^{-1} + A_2^{-1}$. In particular, if $f_1$ is more log-convex than $g_{A_1}$, then without any further assumptions on $f_2$, $f_1 *f_2$ is also more log-convex than $g_{A_1}$.

Note that Lemma 2.1 (ii) verifies that if $\textbf{f}\in \mathcal {F}_{\textbf{A},\textbf{B}}$ then ${{\,\mathrm{\text {Conv}}\,}}\textbf{f}\in \mathcal {F}_{\textbf{A},\textbf{B}}$. For $A_i = \lambda _i \text {Id}_n$, $\lambda _i > 0$, Lemma 2.1 is proved in [33, Proposition 2.4 and Lemma 6.2].

### Proof

If $f_i$ is more log-concave (respectively, more log-convex) than $g_{A_i}$, then after translation and scaling, clearly $f_i(\frac{x \pm \cdot }{\sqrt{2}})$ is more log-concave (respectively, more log-convex) than $g_{A_i/2}$, and hence their product is more log-concave (respectively, more log-convex) than $g_{(A_1+A_2)/2}$, establishing part (i).

Part (ii) is an immediate consequence of a result of Brascamp–Lieb [12, Theorem 4.3], see [38, Theorem 3.7(b)] for an explicit proof of the log-concave statement. The “in particular" part follows formally by taking $A_2 = \Lambda \cdot \text {Id}_n$ and sending $\Lambda \nearrow +\infty$. To verify this rigorously, if $f_1 = g_{A_1} h_1$ with $h_1$ log-convex then:

$$ e^{\frac{1}{2} \left\langle A_1 x,x \right\rangle } (f_1 *f_2)(x) = \int _{\mathbb {R}^n} h_1(x-y) f_2(y) e^{\left\langle x, A_1 y \right\rangle -\frac{1}{2} \left\langle A_1 y,y \right\rangle } dy . $$

For each *y*, the integrand is log-convex in *x*, and so by Hölder’s inequality the integral is also log-convex in *x*, concluding the proof. □

Let us now go over the steps in [33] and check the required modifications. Step (1) is proved in [33, Theorem 2.1] for $\textbf{A}= \lambda \text {Id}$ and $\textbf{B}= \Lambda \text {Id}$ with $0< \lambda \le \Lambda < \infty$, and the proof, based on compactness by means of the Arzelà–Ascoli theorem, immediately extends to general $0 < \textbf{A}\le \textbf{B}$.

Step (2) is proved in [33, Proposition 2.4] for $\textbf{A}= \lambda \text {Id}$ and $\textbf{B}= \Lambda \text {Id}$ with $0< \lambda \le \Lambda < \infty$. Let us verify the extension to general $0 < \textbf{A}\le \textbf{B}$. Indeed, if $\textbf{f}\in \mathcal {F}_{\textbf{A},\textbf{B}}$, we may assume without loss of generality that $\int _{\mathbb {R}^{n_i}} f_i(x_i) dx_i = 1$. Set

$$ F(x) {:}{=} e^{-\frac{1}{2} \left\langle Q x ,x \right\rangle } \Pi _{i=1}^m f_i(x_i)^{c_i} , $$

and compute using the change of variables $u = \frac{x+y}{\sqrt{2}}$ and $v = \frac{x-y}{\sqrt{2}}$:

$$\begin{aligned}&\mathcal{B}\mathcal{L}(\textbf{f})^2 = \left( \int _{\mathbb {R}^N} F(x) dx\right) ^2 = \int _{\mathbb {R}^N} \int _{\mathbb {R}^N} F(u) F(v) du dv \\&= \int _{\mathbb {R}^N} \int _{\mathbb {R}^N} e^{-\frac{1}{2} \left\langle Q u,u \right\rangle } e^{-\frac{1}{2} \left\langle Q v,v \right\rangle } \Pi _{i=1}^m \left( f_i(u_i) f_i(v_i)\right) ^{c_i} du dv \\&= \int _{\mathbb {R}^N} e^{-\frac{1}{2} \left\langle Q x,x \right\rangle } \int _{\mathbb {R}^N} e^{-\frac{1}{2} \left\langle Q y,y \right\rangle } \Pi _{i=1}^m \left( f_i\left( \frac{x_i+y_i}{\sqrt{2}}\right) f_i\left( \frac{x_i-y_i}{\sqrt{2}}\right) \right) ^{c_i} dy dx . \end{aligned}$$

By Lemma 2.1 (i), for each $x \in \mathbb {R}^{N}$, $\textbf{f}_x{:}{=} \left( f_i\left( \frac{x_i+\cdot }{\sqrt{2}}\right) f_i\left( \frac{x_i-\cdot }{\sqrt{2}}\right) \right) _{i=1,\ldots ,m}$ belongs to $\mathcal {F}_{\textbf{A},\textbf{B}}$, and so we may proceed as follows:

$$\begin{aligned}&\ge I_{\textbf{A},\textbf{B}} \int _{\mathbb {R}^N} e^{-\frac{1}{2} \left\langle Q x,x \right\rangle } \Pi _{i=1}^m \left( \int _{\mathbb {R}^{n_i}} f_i\left( \frac{x_i+y_i}{\sqrt{2}}\right) f_i\left( \frac{x_i-y_i}{\sqrt{2}}\right) dy_i\right) ^{c_i} dx \\&= I_{\textbf{A},\textbf{B}} \int _{\mathbb {R}^N} e^{-\frac{1}{2} \left\langle Q x,x \right\rangle } \Pi _{i=1}^m {{\,\mathrm{\text {Conv}}\,}}f_i(x_i)^{c_i} dx = \mathcal {I}_{\textbf{A},\textbf{B}} \, \mathcal{B}\mathcal{L}({{\,\mathrm{\text {Conv}}\,}}\textbf{f}) , \end{aligned}$$

where the first equality follows by definition of ${{\,\mathrm{\text {Conv}}\,}}f_i$ and a change of variables, and the second since ${{\,\mathrm{\text {Conv}}\,}}f_i$ all integrate to 1.

Step (3) is proved in [33, Theorem 2.3] for $\textbf{A}= \lambda \text {Id}$ and $\textbf{B}= \Lambda \text {Id}$ with $0< \lambda \le \Lambda < \infty$. Let us verify the extension to general $0 < \textbf{A}\le \textbf{B}$. Indeed, the infimum in the definition of $I_{\textbf{A},\textbf{B}}$ is attained on some minimizing $\textbf{f}\in \mathcal {F}_{\textbf{A},\textbf{B}}$ by step (1). Hence, by step (2),

$$ \mathcal {I}_{\textbf{A},\textbf{B}}^2 = \mathcal{B}\mathcal{L}(\textbf{f})^2 \ge \mathcal {I}_{\textbf{A},\textbf{B}} \mathcal{B}\mathcal{L}({{\,\mathrm{\text {Conv}}\,}}\textbf{f}) . $$

By Lemma 2.1 (ii), ${{\,\mathrm{\text {Conv}}\,}}\textbf{f}\in \mathcal {F}_{\textbf{A},\textbf{B}}$, and hence

$$ \mathcal {I}_{\textbf{A},\textbf{B}} \ge \mathcal{B}\mathcal{L}({{\,\mathrm{\text {Conv}}\,}}\textbf{f}) \ge \mathcal {I}_{\textbf{A},\textbf{B}} , $$

so ${{\,\mathrm{\text {Conv}}\,}}\textbf{f}$ is also a minimizer. Iterating this, we deduce that $\mathcal {I}_{\textbf{A},\textbf{B}} = \mathcal{B}\mathcal{L}({{\,\mathrm{\text {Conv}}\,}}^k \textbf{f})$ with ${{\,\mathrm{\text {Conv}}\,}}^k \textbf{f}\in \mathcal {F}_{\textbf{A},\textbf{B}}$ for all k≥1. Invoking the Central Limit Theorem as in [33], it follows that each ${{\,\mathrm{\text {Conv}}\,}}^k f_i$ converges in $L^1(\mathbb {R}^{n_i})$ and almost everywhere as $k \rightarrow \infty$ to a centered Gaussian $\frac{1}{Z_i} g_{C_i} \in \mathcal {F}_{\textbf{A},\textbf{B}}$. By Fatou’s lemma, denoting $\textbf{g}= (g_{C_1},\ldots ,g_{C_m})$,

$$ \mathcal {I}_{\textbf{A},\textbf{B}} = \lim _{k \rightarrow \infty } \mathcal{B}\mathcal{L}({{\,\mathrm{\text {Conv}}\,}}^k \textbf{f}) \ge \mathcal{B}\mathcal{L}(\textbf{g}) \ge \mathcal {I}^\mathcal {G}_{\textbf{A},\textbf{B}}. $$

Since the converse inequality is trivial, we verify that $\mathcal {I}_{\textbf{A},\textbf{B}} = \mathcal {I}^\mathcal {G}_{\textbf{A},\textbf{B}}$.

Step (4) is proved in [33, Lemma 2.7] for $\textbf{Q}= \lambda _0 \text {Id}$, $\lambda _0 \ge 0$. Let us verify the extension to general $\textbf{Q}\ge 0$. Given $\textbf{f}\in \mathcal {F}_\textbf{Q}$, the idea is to define

$$ \textbf{f}_{\lambda ,\Lambda } {:}{=} ((f_i g_{2 \lambda \text {Id}_{n_i}}) *g_{\Lambda \text {Id}_{n_i}})_{i=1,\ldots ,m}. $$

Note that $f_i g_{2 \lambda \text {Id}_{n_i}}$ is more log-concave than $g_{Q_i + 2 \lambda \text {Id}_{n_i}}$, and so by Lemma 2.1 (ii), by choosing Λ large enough (in a manner depending only on $\textbf{Q}$ and λ), $(f_i g_{2 \lambda \text {Id}_{n_i}}) *g_{\Lambda \text {Id}_{n_i}}$ is more log-concave than $g_{Q_i + \lambda \text {Id}_{n_i}}$, and in addition more log-convex than $g_{\Lambda \text {Id}_{n_i}}$. Consequently, $\textbf{f}_{\lambda , \Lambda } \in \mathcal {F}_{\textbf{Q}+ \lambda \text {Id}, \Lambda \text {Id}}$, from whence the argument in [33] may be repeated verbatim.

Combining steps (3) and (4), we conclude that:

$$ I_\textbf{Q}= \lim _{\lambda \rightarrow 0^+} \lim _{\Lambda \rightarrow \infty } I_{\textbf{Q}+ \lambda \text {Id}, \Lambda \text {Id}} = \lim _{\lambda \rightarrow 0^+} \lim _{\Lambda \rightarrow \infty } I^\mathcal {G}_{\textbf{Q}+ \lambda \text {Id}, \Lambda \text {Id}} \ge I^\mathcal {G}_{\textbf{Q}} . $$

But since $I^\mathcal {G}_{\textbf{Q}} \ge I_\textbf{Q}$ we must have equality, concluding the proof of Theorem 1.2.

## Comparison with Royen’s approach

Recall that *X* in Theorem 1.4 denotes a centered Gaussian random vector in $\mathbb {R}^N$, whose (positive-definite) covariance matrix Σ is written in block form as

3.1 $$\begin{aligned} \Sigma = \begin{pmatrix} \Sigma _1 &  \Sigma _o \\ \Sigma _o^* &  \Sigma _2 \end{pmatrix} \end{aligned}$$

with $\Sigma _i = P_i \Sigma P_i^*$, i=1,2, where $P_i x = x_i$ for $x = (x_1,x_2) \in \mathbb {R}^{n_1} \oplus \mathbb {R}^{n_2} = \mathbb {R}^N$.

Royen’s proof that $\mathbb {E}{\textbf {1}}_{K_1}(P_1 X) {\textbf {1}}_{K_2}(P_2 X) \ge \mathbb {E}{\textbf {1}}_{K_1}(P_1 X) \mathbb {E}{\textbf {1}}_{K_2}(P_2 X)$ seems to be rather specific to the case that $K_i$ are cubes (or rectangles), but on the other hand applies to the case when $(X_i^2)_{i=1,\ldots ,N}$ is distributed according to more general Gamma distributions. On the other hand, our proof remains unchanged if one replaces $\textbf{1}_{K_i}$ by any two other even log-concave functions $f_i: \mathbb {R}^{n_i} \rightarrow \mathbb {R}_+$, but is restricted to the Gaussian case (and of course *a posteriori*, the correlation inequality ultimately holds for any two even quasi-concave functions $f_i$). Let us now take a more in depth look at the advantages of each approach.

### The equality case in Theorem 1.4

In [36], Royen in fact showed that equality occurs in Theorem 1.4 if and only if $(X_1,\ldots ,X_{n_1})$ and $(X_{n_1+1},\ldots ,X_N)$ are independent, or equivalently, if and only if $\Sigma _o = 0$. Let us give an alternative proof of this following our approach.

#### Proposition 3.1

Equality occurs in Theorem 1.4 if and only if $\Sigma _o = 0$.

#### Proof

The “if" direction is trivial, so assume that we have equality in Theorem 1.4. This means that the left- and right-hand sides of (1.2) coincide and are equal to 1, and so employing the notation used in Section 2, this means that $f_i = {\textbf {1}}_{[-1,1]^{n_i}} g_{\Sigma _i^{-1}}$ is an extremizer of $\mathcal{B}\mathcal{L}(\textbf{f})$ over $f \in \mathcal {F}_{\textbf{Q}}$ with $\textbf{Q}= (\Sigma _1^{-1},\Sigma _2^{-1})$. By step (3) from Section 2, there also exist centered Gaussian extremizers $\textbf{g}= (g_1,g_2) \in \mathcal {G}_{\textbf{Q}}$ with $\mathcal{B}\mathcal{L}(\textbf{g}) = \mathcal{B}\mathcal{L}(\textbf{f}) = 1$. Since the covariance of $f_i$ does not change under each iteration of the self-convolution operator ${{\,\mathrm{\text {Conv}}\,}}^{k} f_i$, this is also preserved in the limit as $k \rightarrow \infty$ and hence $g_i$ and $f_i$ have the same covariance matrices. Consequently, $g_i = g_{\Sigma _i^{-1} + A_i}$ for some positive-definite $A_i > 0$, i=1,2 (as opposed to merely positive semi-definite). Since the ratio in (1.4) is equal to 1 for these particular $A_i$, we have equality in Fischer’s inequality, and we deduce from Lemma 1.5 and (1.6) that $\sqrt{A_1} \Sigma _o \sqrt{A_2} = 0$. But since $A_i$ are positive-definite, we conclude that $\Sigma _o =0$, as asserted. □

### Original argument

Our original proof that the infimum in (1.3) is equal to 1 proceeded by rewriting (1.3) as:

3.2 $$\begin{aligned} \inf _{A_1,A_2 \ge 0} \left( \frac{\det (\text {Id}_{N} + (A_1 \oplus A_2) (\Sigma _1 \oplus \Sigma _2))}{ \det (\text {Id}_{N} + (A_1 \oplus A_2) \Sigma )}\right) ^{\frac{1}{2}} , \end{aligned}$$

and employing the following:

#### Lemma 3.2

Let Σ be a N×N positive-definite matrix, given in block form by (3.1). Denote

3.3 $$\begin{aligned} \Sigma ^{(s)} {:}{=} (1-s) (\Sigma _1 \oplus \Sigma _2) + s \Sigma = \begin{pmatrix} \Sigma _1 &  s \Sigma _o \\ s \Sigma _o^* &  \Sigma _2 \end{pmatrix} ~,~ s \in [0,1] . \end{aligned}$$

Then for all $n_i \times n_i$ positive semi-definite matrices $A_i \ge 0$, i=1,2, the function $[0,1] \ni s \mapsto \det (\text {Id}_N + (A_1 \oplus A_2) \Sigma ^{(s)})$ is monotone non-increasing. In particular, the infimum in (3.2) is 1.

By choosing an appropriate orthonormal basis, one easily reduces to the case where $A_i$ are diagonal. In that case, Lemma 3.2 was established by Royen for his proof of Theorem 1.4 in [36]. Curiously, in [36], the diagonal elements of $A_1 \oplus A_2$ represent the coefficients of the Laplace transform of the distribution of $(X_i^2/2)_{i=1,\ldots ,N}$, whereas in our case, the $A_i^{-1}$’s correspond to the covariances of the Gaussians which potentially saturate the correlation inequality. For completeness, we reproduce the detailed proof in [28] following [36] below.

#### Proof of Lemma 3.2

Denote $[N] = \{1,\ldots ,N\}$, and for J⊂[N], denote by $B_J$ is the principal minor of an N×N matrix *B* with rows and columns in *J*. Also denote |B|=det(B) for brevity. For any pair of orthogonal transformations $U_i \in O(n_i)$,

$$ |\text {Id}_N + (A_1 \oplus A_2) \Sigma ^{(s)}| = |\text {Id}_N + (U_1^{-1} A_1 U_1 \oplus U_2^{-1} A_2 U_2) (U_1 \oplus U_2)^{-1} \Sigma ^{(s)} (U_1 \oplus U_2) | . $$

Choosing appropriate $U_i$ and redefining $A_i$, $\Sigma _i$ and Σ, we may assume that $A_i$ are diagonal. Denote $A{:}{=} A_1 \oplus A_2$, a diagonal matrix. Then by [28, Lemma 5 (i)]:

$$ |\text {Id}_N + A \Sigma ^{(s)}| = 1 + \sum _{\emptyset \ne J \subset [N]} |(A \Sigma ^{(s)})_J| = 1 + \sum _{\emptyset \ne J \subset [N]} |A_J| |\Sigma ^{(s)}_J| . $$

Since A≥0 and hence $|A_J| \ge 0$, it is enough to show that $[0,1] \ni s \mapsto |\Sigma ^{(s)}_J|$ is non-increasing for each $\emptyset \ne J \subset [N]$. Denote $J_1 = J \cap [n_1]$ and $J_2 = J \setminus [n_1]$. If $J_1 = \emptyset$ or $J_2 = \emptyset$ then $\Sigma ^{(s)}_J \equiv \Sigma _J$ remains constant. Otherwise, write:

$$ \Sigma ^{(s)}_J = \begin{pmatrix} \Sigma _{J_1} &  s \Sigma _{J_1 J_2} \\ s \Sigma _{J_2 J_1} &  \Sigma _{J_2} \end{pmatrix} , $$

and observe by [28, Lemma 5 (ii)] that:

$$ |\Sigma ^{(s)}_J| = |\Sigma _{J_1}| |\Sigma _{J_2}| |\text {Id}_{J_1} - s^2 M| ~,~ M {:}{=} \Sigma _{J_1}^{-1/2} \Sigma _{J_1 J_2} \Sigma _{J_2}^{-1} \Sigma _{J_2 J_1} \Sigma _{J_1}^{-1/2} , $$

with $0 \le M \le \text {Id}_{J_1}$. It follows that $[0,1] \ni s \mapsto |\Sigma ^{(s)}_J|$ is non-increasing, concluding the proof. □

### Monotonicity

Royen actually showed that $[0,1] \ni s \mapsto \mathbb {E}\textbf{1}_{[-1,1]^N}(X^{(s)})$ is monotone non-decreasing, where $X^{(s)}$ denotes a centered Gaussian random vector with covariance $\Sigma ^{(s)}$ defined in (3.3). While our proof (employing Lemma 3.2 instead of Lemma 1.5) only shows that this is lower-bounded by a monotone non-decreasing function, it elucidates the reason for the correlation – the worst case is given by $\mathbb {E}g_{A_1 \oplus A_2}(X^{(s)})$, which is explicitly computable and turns out to be monotone in *s*.

Royen’s result is equivalent to the statement that $[0,1] \ni s \mapsto \mathbb {E}f_1(Y_s) f_2(Z_s)$ is monotone non-decreasing for any even quasi-concave functions $f_1,f_2: \mathbb {R}^n \rightarrow \mathbb {R}_+$ and a family of centered Gaussian random vectors $(Y_s,Z_s)$ in $\mathbb {R}^{n} \times \mathbb {R}^n$ having covariance matrix

3.4 $$\begin{aligned} \begin{pmatrix} \Sigma _{YY} &  s \Sigma _{YZ} \\ s \Sigma _{YZ}^* &  \Sigma _{ZZ} \end{pmatrix} . \end{aligned}$$

Indeed, by a standard layer-cake decomposition of $f_1$ and $f_2$ into their super-level sets, the claim reduces to the case when $f_i = {\textbf {1}}_{K_i}$ with $K_i$ an origin-symmetric convex set in $\mathbb {R}^n$. To see the monotonicity in that case, approximate $K_i$ by an origin-symmetric polytope $\cap _{j=1}^{n_i} \{ x \in \mathbb {R}^n: |\langle x,a^i_j \rangle | \le 1\}$, and arrange $\{a^i_j\}_{j=1,\ldots ,n_i}$ as the rows of an $n_i \times n$ matrix $A_i$. Treating all random vectors as column vectors, define the centered Gaussian random-vector $X^{(s)} = (A_1 Y_s + \epsilon G_1, A_2 Z_s + \epsilon G_2)$ in $\mathbb {R}^N$, where $N = n_1+n_2$, and $G_i$ is a standard Gaussian random vector in $\mathbb {R}^{n_i}$ which is independent of all other random vectors. It follows that the (non-degenerate) covariance matrix of $X^{(s)}$ is given by

$$ \begin{pmatrix} A_1 \Sigma _{YY} A_1^* + \epsilon ^2 \text {Id}_{n_1} &  s A_1 \Sigma _{YZ} A_2^* \\ s A_2 \Sigma _{YZ}^* A_1^* &  A_2 \Sigma _{ZZ} A_2^* + \epsilon ^2 \text {Id}_{n_2} \end{pmatrix} . $$

Royen’s result implies that $[0,1] \ni s \mapsto \mathbb {E}\textbf{1}_{[-1,1]^N}(X^{(s)})$ is monotone, and the monotonicity is preserved in the limit after refining the approximation of the $K_i$’s and sending $\epsilon \rightarrow 0$, thereby establishing the monotonicity of $[0,1] \ni s \mapsto \mathbb {E}\textbf{1}_{K_1}(Y_s) \textbf{1}_{K_2}(Z_s)$.

It is worthwhile noting that this monotonicity has a useful consequence for the Ornstein–Uhlenbeck semi-group $P_t$ associated to the standard Gaussian measure $\gamma ^n$ on $\mathbb {R}^n$, defined for (say) $f \in L^2(\gamma ^n)$ as

$$ P_t f(y) {:}{=} \mathbb {E}f (e^{-t} y + \sqrt{1-e^{-2t}} Z_0) ~,~ Z_0 \sim \gamma ^n ~,~ t \in [0,\infty ]~,~ y \in \mathbb {R}^n . $$

Note that $P_0 f = f$ and $P_\infty f \equiv \int f d\gamma ^n$. Consequently, the Gaussian Correlation Inequality may be equivalently reformulated as stating that for any even quasi-concave functions $f_1,f_2: \mathbb {R}^n \rightarrow \mathbb {R}_+$, one has:

$$ \int f_1 P_0 f_2 d\gamma ^n \ge \int f_1 P_\infty f_2 d\gamma ^n . $$

Royen’s argument actually implies the stronger statement that $[0,\infty ] \ni t \mapsto \int f_1 P_t f_2 d\gamma ^n$ is monotone non-increasing; when n=2, this was previously established by Pitt [35]. To see this, note that

$$ \int f_1 P_t f_2 d\gamma ^n = \mathbb {E}(f_1(Y) f_2 (Z_s)) ~ ,~ Z_s = s Y + \sqrt{1-s^2} Z_0 ~,~ s = e^{-t} , $$

where *Y* and $Z_0$ are independent standard Gaussian random vectors in $\mathbb {R}^n$. Consequently, the covariance matrix of the random vector $(Y,Z_s)$ in $\mathbb {R}^{n} \times \mathbb {R}^n$ is of the form (3.4) with $\Sigma _{YY} = \Sigma _{YZ} = \Sigma _{ZZ} = \text {Id}_n$, and the monotonicity follows.

In contrast, we do not know how to obtain the full monotonicity of $P_t$ using the inverse Brascamp–Lieb approach.

### Subsequent developments

After posting and submitting our work, Nakamura and Tsuji noted that there is actually no need to assume that the functions $f_i = g_{Q_i} h_i$ in Theorem 1.2 are even, and that in order to run the Central Limit argument in step (3) of the proof in Section 2, it is only necessary that their barycenter is at the origin. After treating various additional technicalities which arise, they were able to show in [34] that:

3.5 $$\begin{aligned} \gamma ^n(K \cap L) \ge \gamma ^n(K) \gamma ^n(L) \end{aligned}$$

for all convex $K,L \subset \mathbb {R}^n$ whose Gaussian barycenter is at the origin (“centered"), including a characterization of the equality case. In the origin-symmetric case, a characterization of the equality case can also be deduced from a stability version of (3.5) obtained in [21, Theorem 21 and Proposition 23]. We refer to [34] for further extensions of (3.5) to the case when the Gaussian barycenters of *K* and *L* coincide (but differ from 0), and for a correlation result for more than two centered convex sets $K_1,\ldots ,K_m$.

In contrast, it seems that Royen’s approach is inherently restricted to origin-symmetric sets and even functions, because the first step in his proof is to consider the squared random vector $(X_i^2)_i$, and its distribution can only capture information on the sigma-algebra generated by events of the form $X_i \in [-r_i,r_i]$.

## Data Availability

No datasets were generated or analysed during the current study.
